# Light modulates oscillatory alpha activity in the occipital cortex of totally visually blind individuals with intact non-image-forming photoreception

**DOI:** 10.1038/s41598-018-35400-9

**Published:** 2018-11-16

**Authors:** Gilles Vandewalle, Markus J. van Ackeren, Véronique Daneault, Joseph T. Hull, Geneviève Albouy, Franco Lepore, Julien Doyon, Charles A. Czeisler, Marie Dumont, Julie Carrier, Steven W. Lockley, Olivier Collignon

**Affiliations:** 1 Functional Neuroimaging Unit, University of Montréal Geriatric Institute, Montréal, Québec Canada; 2 Center for Advanced Research in Sleep Medicine, Hôpital du Sacré-Cœur de Montréal, Montréal, Québec Canada; 3 GIGA-Institute - Cyclotron Research Centre/In Vivo Imaging Unit, University of Liège, Liège, Belgium; 4 Center for Mind and Brain Science, University of Trento, Trento, Italy; 5 Division of Sleep and Circadian Disorders, Departments of Medicine and Neurology, Brigham and Women’s Hospital, Boston, Massachusetts, USA; 6 Division of Sleep Medicine, Harvard Medical School, Boston, Massachusetts, USA; 7 Centre de Recherche en Neuropsychologie et Cognition (CERNEC), Université de Montréal, Montréal, Québec Canada; 8 Institute for research in Psychology (IPSY) and Neuroscience (IoNS), Université catholique de Louvain (UcL), Louvain-la-Neuve, Belgium

## Abstract

The discovery of intrinsically photosensitive retinal ganglion cells (ipRGCs) marked a major shift in our understanding of how light information is processed by the mammalian brain. These ipRGCs influence multiple functions not directly related to image formation such as circadian resetting and entrainment, pupil constriction, enhancement of alertness, as well as the modulation of cognition. More recently, it was demonstrated that ipRGCs may also contribute to basic visual functions. The impact of ipRGCs on visual function, independently of image forming photoreceptors, remains difficult to isolate, however, particularly in humans. We previously showed that exposure to intense monochromatic blue light (465 nm) induced non-conscious light perception in a forced choice task in three rare totally visually blind individuals without detectable rod and cone function, but who retained non-image-forming responses to light, very likely via ipRGCs. The neural foundation of such light perception in the absence of conscious vision is unknown, however. In this study, we characterized the brain activity of these three participants using electroencephalography (EEG), and demonstrate that unconsciously perceived light triggers an early and reliable transient desynchronization (i.e. decreased power) of the alpha EEG rhythm (8–14 Hz) over the occipital cortex. These results provide compelling insight into how ipRGC may contribute to transient changes in ongoing brain activity. They suggest that occipital alpha rhythm synchrony, which is typically linked to the visual system, is modulated by ipRGCs photoreception; a process that may contribute to the non-conscious light perception in those blind individuals.

## Introduction

While vision is the best understood sensory system in the human brain, we are just beginning to understand the neuronal pathways mediating the ‘non-image-forming’ effects of ocular light exposure^1,2^. Evidence for non-rod, non-cone retinal photoreceptors had been suspected in mice, mole rats and humans^3–9^, before the discovery of intrinsically photosensitive retinal ganglion cells (ipRGCs) was reported in animals at the turn of the century^10–12^. These retinal ganglion cells express the photosensory opsin, melanopsin, which is maximally sensitive to short-wavelength blue light (~480 nm). IpRGCs are the primary photoreception channel mediating non-image-forming functions of light including entrainment of the circadian clock, suppression of the pineal hormone melatonin, and pupillary constriction^1,13–15^. IpRGC light signaling has also been strongly suggested to acutely enhance human alertness and cognitive brain functions^16–18^. Melanopsin-driven intrinsic responses of ipRGCs are more sluggish than the response of the classical photoreceptors^19^. IpRGCs also receive incoming signals from rods and cones, however, so their response output is phasic^1,20^. This photic response then propagates to non-image-forming brain areas, including hypothalamic nuclei notably involved in circadian rhythm generation and sleep-wake regulation^21,22^. IpRGCs also project to brain areas that primarily mediate vision in rodents and primates, such as the lateral geniculate nucleus of the thalamus^20,22^. It has been suggested that ipRGCs contribute to basic vision (e.g. low-spatial frequency contrast, brightness detection, and adjustment of the sensitivities of rods and cones) in humans and rodents^23–29^. Similarly to animal models, many human non-image-forming responses to light are more sensitive to shorter wavelength (blue) light suggesting a role of ipRGCs in these responses^5,16,30–33^. Isolating the independent role of ipRGCs apart from those of rods, cones and other non-photosensitive RGCs can be achieved in animals by knocking down, amplifying, or modifying part of the photoreception system^26,34^. Recently, new psychophysical techniques have also been developed in order to target melanopsin separately from the cones (e.g. silent substitution using metameric lights)^18,24,35–38^. However, the specific role of ipRGCs in non-image-forming functions remains difficult to isolate in human subjects who have intact retinal (rod/cone) function.

To investigate ipRGCs in isolation, we studied a rare and small group (n = 9) of totally visually blind human individuals who despite absence of detectable rod and cone function, retained non-image-forming responses to light, very likely via ipRGCs. These blinds individuals have been reported to exhibit normal 24-hour circadian rhythms, light-induced melatonin suppression, pupil constriction and/or circadian phase resetting in response to nighttime ocular light exposure^3,19,39–41^. The etiology of blindness of most of these individuals was due to retinitis or retinopathy and a neuro-ophthalmologic exam confirmed individuals’ reports of having no conscious light perception suggesting the absence of rod and cone function^41^. The implication of ipRGCs is supported by the observation that ocular exposure to light in the shorter wavelength light range (blue; ~470 nm) compared to light in longer wavelength ranges (>500 nm), was more efficient in triggering an alerting or pupil response^19,40^. Furthermore, when exposed to blue light during a cognitively challenging task as compared to while in darkness, these atypical blind individuals showed enhanced activation in a widespread subcortical and cortical network, including the occipital cortex (using functional magnetic resonance imaging – fMRI)^17^. Altogether, these results support the notion that these blind individuals provide a unique human model system to study the role of ipRGCs in the absence of conscious vision.

Critically, these rare individuals present the ability to non-randomly detect the presence or absence of light in a two-alternative choice forced task (2AFC) indicating some degree of light perception in the absence of conscious vision [n = 1^40^; n = 3^17^]. The brain mechanisms involved in this non-conscious light perception are unknown. Cortical oscillations in the alpha range (8–14 Hz) may provide important insights to address this issue. In sighted individuals, alpha oscillations show transient responses to visual stimulation, which emerge in the order of milliseconds to seconds. These oscillations are thought to reflect changes in visual attention allocation and are typically observed *locally* as a *transient decrease* in alpha power, or a transient desynchronization, over the area of the brain that processes the stimulus presented^42–46^. Interestingly, recent evidence suggests that these more transient oscillatory changes could be modulated by prior exposure to bright blue light, which likely implicate input from ipRGCs^47,48^. Exposure to blue light at night also *increases* high alpha power in sighted individuals (~10–12 Hz)^32,49,50^, as well as in a blind individual with presumably intact ipRGCs (~8–10 Hz)^40^. However, these *sustained* changes in alpha power are typically reported over the *entire* cortex following prolonged exposure to light and seem to take minutes to tens of minutes to be detected.

Alpha transient responses to light have not been investigated in blind participants who retain non-image-forming responses. Since non-conscious light perception appears somewhat typically visual in these blind individuals, we hypothesized that ocular light exposure would decrease alpha power over the occipital cortex transiently, which would then support the concept that non-conscious light perception arises from a specific impact on visual attention.

To test this hypothesis, EEG was recorded in three totally visually blind individuals who previously exhibited non-image forming responses to light during a forced choice task during which they chose the presence or absence of light non-randomly despite their complete lack of conscious vision^17^. Spectral analysis and EEG source reconstruction computations were performed to quantify short-term (10 s) light-induced changes in alpha rhythm over the occipital cortex task.

## Methods

These data were collected simultaneously with data reported in^17^; the EEG data and analyses reported herein have not been reported previously. Except for EEG data analyses, methods included here were described previously^17^. All experiments were approved by the Comité mixte d’éthique de la recherche du Regroupement Neuroimagerie/Québec. All methods were carried out in accordance with Canada and Québec guidelines and regulations.

### Participants

Three totally visually blind individuals participated [1 female/2 males, range 60–67 years; see Table 1 for detailed characteristics, previously reported in^17^]. Each participant provided written informed consent and received financial compensation for their participation. Each participant reported having no conscious light perception, but each previously exhibited light-induced melatonin suppressions to 6.5 h ocular exposure to white^39–41^. A neuro-ophthalmologic examination was conducted in each participant. A Visual Evoked Potential (VEP) test was performed in Participants 1 and 2, and an electroretinogram (ERG) was performed in Participant 3; no detectable activation was observed. Participant 1 had miotic pupil bilaterally, while participant 3 had no clearly distinguishable pupil. Only participant 2 exhibited pupil constriction after prolonged (>10 s) light exposure^19^. A fundoscopic examination in Participants 1 and 2 confirmed atrophy of the retinal pigment epithelium, with thinning of retinal vessels and bone spicule pigmentation.

**Table 1:** Participants characteristics.

	Participant 1	Participant 2	Participant 3
Age	67	60	66
Sex	Female	Male	Male
Body Mass index (BMI)	27.3	30.0	27.4
Cause of blindness	Retinitis Pigmentosa	Retinitis Pigmentosa	Retinopathy of Prematurity
Years of total blindness	10	25	66
Chronotype (Horne & Ostberg, 1976)	62 - Moderate morning type	72 - Moderate morning type	68 - Moderate morning type
Sleep disturbances (from 0 = lowest, to 21 = highest) (Buysse *et al*., 1989)	3	10	4
Daytime propensity to fall asleep (Johns, 1991)	1	4	16
Last confirmation of light – induced melatonin suppression	in 2008	in 2006	in 2002
Previous standard ERG examination (no detectable signal)	No	No	in 1994
Previous standard VEP examination (no detectable signal)	in 2008	in 2006	No
Pupil response to prolonged light exposure (>5 s)	No	Yes	No
Participated in a published study	^41^	^19, 40, 41^	^39, 41^

A more exhaustive description of participants’ characteristics is provided in^17^.

### Experimental design

The experiment was conducted in Montreal in October and November 2009. It is important to stress that the very rare participants involved in the study came for various places in Canada and US and were involved in 2 days of intense testing involving: psychophysics, EEG, fMRI and pupillometry. Since the participants were relatively senior (60, 66 and 67 y at the time of testing), we had to find the best trade-off between carrying out as many tests as possible (including control ones) without creating cognitive overload, stress and fatigue. Our goal was to evaluate whether all three participants would be able to non-randomly detect the presence or absence of light while they underwent EEG recording, in addition to separate EEG and fMRI testing as reported in^17^. For this purpose, we decided to concentrate on the condition that previously provided successful non-conscious vision, namely the blue light condition^40^.

Participants maintained a regular sleep schedule for 7 days before the experiment (verified using sleep logs; the remained on the clock of their original time zone during the entire experiment). The tests were conducted in the afternoon, 6.5 h after habitual wake up time, in an electromagnetically shielded and sound attenuated room in complete darkness. Participants were blindfolded for 1 h upon arrival. The light source covered 120 × 85 degrees of visual angles and was elicited via an array of 48 blue light emitting diodes [LED, peak = 465 nm, Full Width at Half Maximum (FWHM) = 27 nm; spectrum assessed with Lightspex - GretagMacbeth, New Windsor, NY] behind a 21 × 11 cm^2^ diffusion glass with ultraviolet and infrared filters. Photon flux at corneal level was 9.7 × 10^14^ photons/cm^2^/s (see Table 2 for detailed light characteristics). The light device produced no perceptible sounds or temperature change. The light source was controlled via a computer code that pseudo-randomly turned it on or off, without any intervention from the experimenter. The experimenter was in another room and could see the participants and light via video camera and hear the verbal response from the participants. The experimenter could, however, not communicate with the participant once data acquisition was started. Throughout the EEG protocol, participants’ gaze was monitored with an infrared camera to ensure that their eyes remained open.

**Table 2:** Light characteristic.

Peak wavelength (nm)	465
Full width at half maximum (nm)	27
Photopic illuminance (lux)	283
Irradiance (µW/cm^2^)	414
Photon flux (ph/cm^2^ s)	9.97 × 10^14^
Spectrally weighted irradiance (µW/cm^2^):	
- Cyanopic (S cone) stimulation	249.86
- Melanopic stimulation	332.55
- Rhodopic stimulation	275.54
- Chloropic (M cone) stimulation	153.60
- Erythropric (L cone) stimulation	84.83

All values were determined based on the irradiance toolbox published in^1^.

Brief, intermittent blue-light flashes (duration = 500 ms, ISI = 1600–1800ms) were administered during four blocks of 200 trials (800 trials in total) to record any visually evoked potentials (VEP). No EEG responses could be detected confirming previous assessments in these participants and further reinforcing the notion these participants lack residual rod and cone function [see]^17^. EEG recordings were also acquired during a two-alternative forced choice task (2AFC). Each trial lasted 20 s during which blue light was pseudo-randomly presented during the first or last 10 s (10 s light ON and OFF). Each trial started with a pre-recorded auditory cue (“start”) and ended with another pre-recorded auditory cue (“end”). A brief auditory pure tone (500 ms, 1000 Hz) indicated the middle of the trial. After each trial, the participant was asked to state whether the light stimulus was presented during the first or second half of the trial both orally and by pressing a key. Participants were presented with 40 trials in total resulting in 80 segments, half with and half without light stimulus. Only the overall performance on the task is available for Participant 1, (i.e., responses to individual trials were not recorded due to a scripting error), however, despite this, Participant 1 provided the highest number of correct “guesses” about the presence of the light stimulus (95% of correct guesses) compared to the other two participants, such that participant 1 would have had only 2 incorrected guesses to contrast with the 38 correct ones, precluding any reliable analyses between EEG and behavioral measures at the individual trial level.

### EEG data acquisition and preprocessing

EEG was recorded continuously from 40 Ag-AgCl electrodes on an extended 10–20 system (Neurosoft Inc., Sterling, VA) using a sampling rate of 1000 Hz. The signal was filtered online using a band-pass filter (0.1–100 Hz) and referenced to the algebraic average of the two mastoids. Impedances were kept below 5 kΩ. The signal was filtered offline with a low-pass filter with a cut-off at 30 Hz, re-referenced to the algebraic average across electrodes, and down-sampled to 150 Hz. Due to the long duration and limited number of trials of the 2AFC, extended infomax independent component analysis (ICA) was performed with a weight change stop criterion of 10^−7^. Components reflecting ocular artifacts (e.g. eye blinks and lateral saccades) were rejected by MJvA based on visual inspection of topography and time course.

### Spectral analysis

All offline analysis was conducted using Fieldtrip^51^, under Matlab (The Mathworks Inc, Natick, MA). Analysis was performed separately for the 10 s exposure to light (Light ON) and darkness (light OFF). Spectral analysis was applied over the entire 10 s period for frequencies between 5 and 25 Hz using the multitaper method^52^ with 19 Slepian tapers resulting in a frequency smoothing of ±1 Hz. Statistical analysis was performed on the log-transformed power spectra and centered on the alpha band (8–14 Hz) in occipital electrodes (O1, Oz, and O2). Between-trial comparisons for the light-ON *versus* light-OFF segments were conducted for each participant using independent sample t-tests. A cluster permutation approach was used (500 permutations) to control for multiple comparisons using a maximum cluster extent threshold^53^. Permutation tests provide a robust inferential statistic, without assumptions about the shape of the sampling distribution. Furthermore, by selecting a liberal alpha-range (8–14 Hz), the method optimally captures potential variation in the individual alpha peak frequency. To explore the time-course of alpha power modulation in response to light further, a separate time-frequency analysis was performed using 6 cycle Morlet wavelets (100 ms steps, 1 Hz frequency resolution). Here, the frequency-averaged alpha power time course was compared between the light-ON versus light-OFF condition and clustering was performed across time (0–10 s post stimulus onset) and space (electrodes). We want to emphasize that this test followed the initial comparison of the frequency spectra and should, therefore, be considered exploratory.

### Source-level analysis

In line with the recommendations by Gross and collaborators^54^, statistical analysis of the differences between the light-ON and light-OFF condition were performed at the sensor level and subsequent source reconstruction was performed to identify the generators. Source reconstruction was performed using structural MR-images from each of the three blind participants. T1 weighted images (voxel size: 1 × 1 × 1 mm^3^, TR = 2.3 s, TE = 2.91 ms, flip angle = 9°, field of view 265 × 224 mm^2^) were acquired on a 3-T TIM-TRIO MR scanner (Siemens, Erlangen, Germany). Individual T1 MRI images were segmented in cerebrospinal, white matter, grey matter and skull compartments. The white and grey matter brain compartments were used to construct individual head models using the Boundary Element Method (BEM)^55^. Source activity was projected on a regular three dimensional grid (10 × 10 × 10 mm^3^), using a frequency domain beamformer, called Dynamic Imaging of Coherent Sources^56,57^. A regularization of 15% was applied. To identify robust peak differences in the alpha range, statistical maps for the difference between the light-ON versus light-OFF conditions were computed using independent-sample t-tests, using a permutation approach, with a threshold of α = 0.05 (FDR corrected).

## Results

**Figure 1:**
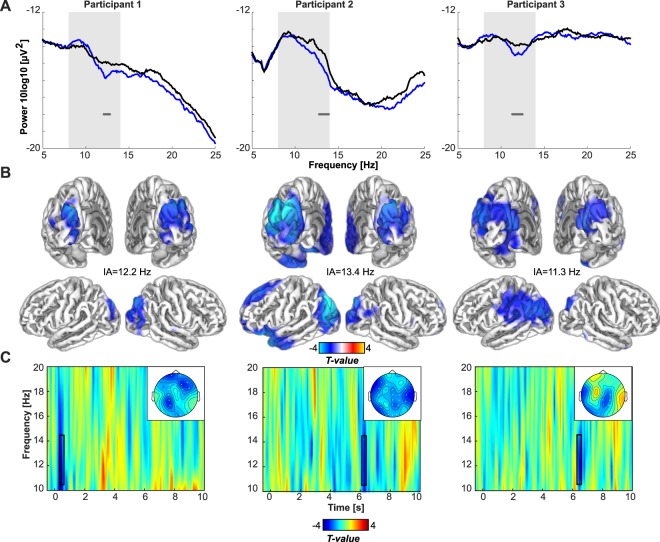
Desynchronization of the occipital alpha/low beta rhythm during 10 second stimulation with blue light in three blind participants with intact non-image-forming photoreception. (**A**) Frequency spectra for the entire 10 s period of blue light (blue), and darkness (black) show differences between conditions in all three participants (horizontal gray bar, p < 0.05, permutation-cluster-corrected for multiple comparisons) for the frequency band of interest (shaded area, 8–14 Hz). (**B**) Source reconstructions depict the difference between conditions (thresholded at p < 0.05, FDR corrected for multiple comparisons), at the individual alpha frequency showing greatest light-induced decrease (see main text). Statistical maps (t-values) of the topographies with the absolute difference between conditions at the individual alpha frequencies show that the spatial extent of the decrease in alpha power is largely restricted to occipital lobe. (**C**) Time-frequency representations illustrate differences between the light-ON versus light-OFF condition in time. Open rectangles highlight the time range of greatest difference (*p* < 0.05, FDR-cluster-corrected for multiple comparisons).

As reported previously^17^, both Participants 1 and 2 showed a very high accuracy in detecting when the blue light stimulus was presented (95%, and 80%, both *p* < 0.001). Participant 3 also showed detection rates different from chance although accuracy was below chance (30%, p = 0.008). A parsimonious explanation for what could appear as a very low performance is that the participant had a sense of the presence/absence of the light stimulus, but he could not correctly attribute the physical presence of the light stimulus to the metacognitive reasoning that the light stimulus was actually presented (see e.g.^58^). EEG spectral analysis of the alpha power difference between the light-ON *versus* light-OFF across occipital electrodes and over the entire 10 s time windows revealed significantly reduced alpha power in all three participants (participant 1: *p* = 0.034; participant 2: *p* = 0.024; participant 3: *p* = 0.028) (Fig. 1A). This sustained difference between conditions was strongest in the upper alpha range [11–14 Hz] for each of the three participants (Participant 1: 12.1–12.8 Hz; Participant 2: 12.8–13.9 Hz; Participant 3: 11.3–12.5 Hz).

**Figure 2:**
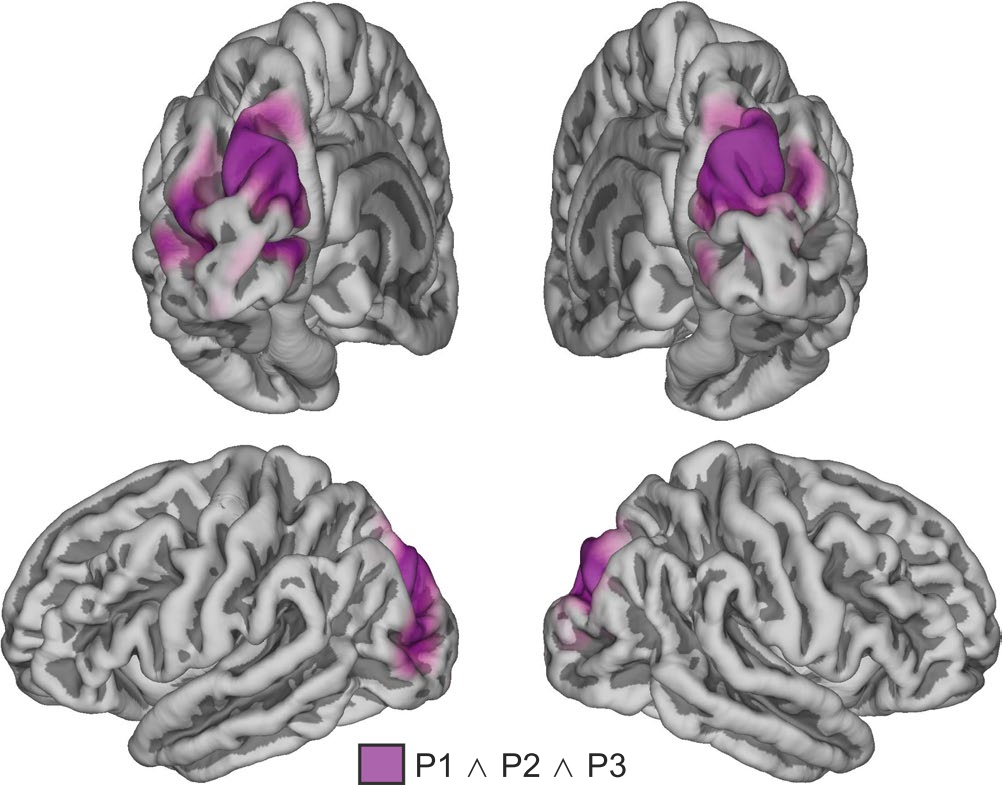
Conjunction analysis across the source statistical maps identified an area in bilateral superior occipital gyrus and cuneus as a significant (p < 0.05) cortical source of the alpha suppression in response to blue light, common to all three participants.

Subsequent source reconstruction was performed (i.e. not restricting to occipital electrodes) on the entire 10 s time window, using the individual peak in alpha power difference between light-ON and light-OFF (participant 1: 12.2 Hz; participant 2: 13.4 Hz; and participant 3: 11.3 Hz; see highlighted peaks on Fig. 1A). Although the analyses were not restricted to the posterior part of the brain, the neural generators of the alpha power decrease during light-ON was localized primarily to occipital cortex in Participants 1 and 2, and to occipito-parietal cortex in Participant 3 (Fig. 1B). In addition, a distinct conjunction analysis, seeking for significant sources common to all participants combining all three statistical maps, revealed an area in bilateral superior occipital gyrus (SOG) (Fig. 2). The light-induced decrease in alpha power observed over occipital sensors seems therefore to be mostly confined to the occipital cortical areas.

Finally, Fig. 1C shows the time-frequency maps for the statistical difference between the light-ON and light-OFF condition for each of the three participants. This exploratory follow-up and distinct analyses on the upper alpha range (11–14 Hz) performed across time and space showed a transient alpha suppression effect. These transient statistically significant changes indicate the time points at which the sustained light-induced reduction in alpha power detected across the 10 s exposure is strongest. Participant 1 showed a broadband significant desynchronization shortly after stimulation onset (400–600 ms, *p* = 0.01), Participant 2 (6200–6400 ms, *p* = 0.012), and Participant 3 (6400–6600 ms, *p* = 0.038) showed significant desynchronization only after several seconds.

## Discussion

Some totally visually blind individuals with preserved non-image-forming photoreception can detect successfully the presence or absence of light in a forced choice task^17,40^. In this study, we investigated the brain mechanisms underlying this non-conscious light perception using EEG data recordings during the 2AFC task. We show that 10 s of light exposure is able to reliably induce a transient and local decrease of alpha power over the occipital cortex in the three participants tested. Although the exact frequency seems to vary slightly across individuals (peak of alpha power decrease between 11.3 Hz and 13.4 Hz), this decrease is more prominent in the higher alpha band between 11 and 14 Hz rather than in the lower alpha range.

Transient alpha oscillations are thought to reflect a sensory gating, or prioritization, mechanism^44,45^. For example, fluctuations in the cortical alpha rhythm have been associated with visual-spatial, sensory, and object-based attention^43,59–61^, including response to a blue-enriched white light^47^. Specifically, transient reduced alpha power typically reflects enhanced neuronal activity, or excitability^62–64^, while transient enhanced alpha power is considered a marker for cortical inhibition^44,45^. For example, if alpha power in a given sensory area is low, participants are more likely to perceive a near-threshold event in the same modality^63,65,66^. If transient reduced alpha power is a marker for increased cortical excitability in visual areas, or indeed a precursor to non-conscious light perception, this could explain why participants made non-random selections about the presence or absence of the blue light during the 2AFC task.

In our view, the source of the effects is most parsimoniously explained by ipRGCs photoreception because (1) these cells are known to be preserved in individuals with outer retinal degeneration such as the 3 participants of the present study^67^, (2) ophthalmological examination confirmed in all 3 participants atrophy of the retinal pigment epithelium and found no detectable functional responses from rods and cones^40^ (cf. Table 1), (3) light effects were more pronounced using 460- to 480-nm monochromatic light as compared with other wavelengths in a previous study including participant 2 of the present study^40^, and (4) recent data show that the dynamics of pupillary constriction in the same participant 2 with outer retinal degeneration was compatible with the exclusive involvement of ipRGCs^19^. Moreover, the fact that no VEP response (see supplementary material) could be observed with 800 flashes of the exact same stimulation demonstrate that it is extremely unlikely that the effect we observed are due to a crude preservation of rods/cones photoreception. Finally, participants had no conscious light perception. Including other wavelengths of light and showing that EEG modulation was stronger with exposure around 480 nm could have strengthened our finding and should be the aim of future investigations, but it would not add to the fact that light modulated EEG activity in individual with no detectable residual outer retina function. Therefore, although we cannot formally exclude a marginal contribution of remaining but yet undetected rod or cones, we provide compelling evidence that the melanopsin-driven photosensitivity of ipRGCs feed to the human visual cortex and trigger some non-conscious light perception.

This assumption is supported by recent data in sighted individuals exposed to metameric light that stimulated melanospin while leaving the other retinal photoreceptor equally stimulated^18,24,35–38^. This type of light stimulation has been used to show that melanopsin-driven ipRGCs output modulate activity of the frontal eye field, a brain region involved in visual attention and ocular motor responses^37^ and of the occipital cortex^35^, extending previous animal research^68^. In addition, metameric light geared toward melanopsin sensitivity indicated that ipRGC output contributed to conscious visual perception of brightness in human^24,35^, but only if the modulation of light spectral composition was relatively slow^35,36^. One could assume that, in blind individuals with intact ipRGCs, brightness is a difficult concept to elaborate on that can be detected in the conscious percept through a forced guess. If true, then this could mean that brightness detection in sighted individual is, at least in part, mediated through a transient change in alpha oscillation over the occipital cortex. This remains to be investigated, using for instance metameric light stimulation^18^. Alternatively, the moment at which alpha is most desynchronized could correspond to moment at which each participants makes, on average, the forced choice decision on the presence/absence of light.

The significant transient change in alpha occipital activity appears to be brief in the time-frequency analysis (~200 ms). The strength of the finding is emphasized by the stringent statistic we applied to our small sample size. Indeed, simple spectral analysis yielded significant alpha reduction on average over the entire 10 s trials. One should consider the time-frequency as an indicator of the moment at which desynchronization is strongest. This moment varied from ~500 ms in participant 1 to ~6 s in the other two participants, which is long, in any case, with respect to classical rod-cone photoreception. This relatively late onset response may be related to the relatively sluggish responses of the intrinsic melanopsin-driven light sensitivity of ipRGCs in the absence of classical photoreceptor inputs^1,11,19,20^.

We can only speculate about the pathways that mediates the occipital effect of light we report. IpRGCs have strong projections to the lateral geniculate nucleus^69^, the principal thalamic relay between the retina and the striate cortex. IpRGCs also project densely to many hypothalamic and non-hypothalamic structures, including the superior colliculus^69^. The superior colliculus project to the extrastriate visual cortex via the pulvinar, such that ipRGCs could affect occipital activity through a colliculo-pulvinar pathway^70^. Interestingly the superior occipital cortex (area 19), which is significantly affected by light across our 3 participants (conjunction analysis), is a heterogeneous peristriate visual area that may contain several visual representations and that receives particularly dense colliculo-pulvinar projections^71,72^. Interestingly also, projections between the pulvinar and extrastriate occipital regions have been implicated in blindsight which is another form of unconscious light perception^73^. One could therefore hypothesize that ipRGC signals reach the occipital cortex through the blindsight pathway. It is however important to note that the phenomena of blindsight has always been interpreted in the context of image-forming pathways linked to rods/cones photoreception and whether ipRGCs play a potential role in blindsight remains purely speculative at this stage. Aside from potential LGN or colliculo-pulvinar pathways one could also envisage a modulation of brain activity through other indirect pathways, for instance via wake-promoting brainstem nuclei^74^.

IpRGCs are considered to mediate at least in part the impact of light on alertness and attention^30^, which is evident through a sustained and global decrease in delta (0.5–4 Hz) and theta (4–8 Hz) power in EEG recordings of spontaneous (i.e. task free) brain activity^49,75^ concomitant to an increase in alpha power^32^. In situation of vigilance (cf.^40^), the impact of light would express at late temporal scale with an increase in alpha power. In contrast, when attending transient stimuli changes, light appears to induce a transient reduction of alpha power at relatively short time-scale. These results therefore bring new information about how ipRGCs can play a prominent role in visual functions that is distinct from the slow fluctuating effects observed in relation to alertness circuits. How related are the perceptual role of ipRGCs and their impact on vigilance and attention requires further investigation. Future research should also use other light wavelengths and intensities, as well as administer light at other times of day to generalize our findings to other light conditions and gain further insight into ipRGC photoreception.

In summary, we demonstrate that short exposure to light, in the order of several seconds, reduces alpha power in occipital cortex in three blind human participants who demonstrate non-conscious light perception. This effect is likely attributed to ipRGCs and could be related to their suggested role in brightness detection in sighted individuals^24,35^. Using a human model of isolated ipRGCs photoreception has proven to be insightful to better comprehend melatonin suppression, pupil and cognition regulation, and circadian phase resetting by light^3,17,19,39,40^. The present results extend these previous results by strengthening the view that ipRGCs photoreception influence the visual system to a much greater extent than previously thought.

## Electronic supplementary material

SUPPLEMENTARY INFORMATION
