# Supplementary material for: Light modulates oscillatory alpha activity in the occipital cortex of totally visually blind individuals with intact non-image-forming photoreception
Source: Sci Rep. 2018 Nov 16;8:16968. doi: 10.1038/s41598-018-35400-9 (PMC6240048; doi:10.1038/s41598-018-35400-9)
Supplement: Supplementary file 1 — SUPPLEMENTARY INFORMATION [file 41598_2018_35400_MOESM1_ESM.pdf]

ONLINE SUPPLEMENTARY INFORMATION

Light modulates oscillatory alpha activity in the occipital cortex of totally visually blind individuals with intact non-image-forming photoreception

Gilles Vandewalle[1,2,3*], Markus J. van Ackeren[4*], Véronique Daneault[1,2], Joseph T. Hull[5,6], Geneviève Albouy[1], Franco Lepore[7], Julien Doyon[1], Charles A. Czeisler[5,6], Marie Dumont[2], Julie Carrier[1,2,7], Steven W. Lockley[5,6], Olivier Collignon[4,7,8]

(1) Functional Neuroimaging Unit, University of Montréal Geriatric Institute, Montréal, Québec, Canada;
(2) Center for Advanced Research in Sleep Medicine, Hôpital du Sacré-Cœur de Montréal, Montréal, Québec, Canada;
(3) GIGA-Institute - Cyclotron Research Centre/In Vivo Imaging Unit, University of Liège, Belgium;
(4) Center for Mind and Brain Science, University of Trento, Italy
(5) Division of Sleep and Circadian Disorders, Departments of Medicine and Neurology, Brigham and Women's Hospital, Boston, Massachusetts, USA;
(6) Division of Sleep Medicine, Harvard Medical School, Boston, Massachusetts, USA;
(7) Centre de Recherche en Neuropsychologie et Cognition (CERNEC), Université de Montréal, Montréal, Québec, Canada;
(8) Institute for research in Psychology (IPSY) and Neuroscience (IoNS), Université catholique de Louvain (UcL), Belgium.

** joint first authors*

Corresponding authors: Gilles Vandewalle, GIGA-Institute - Cyclotron Research Centre/In Vivo Imaging Unit, University of Liège, Bâtiment B30, 8 allée du VI août, B-4000 Liège, Belgium. . Email: gilles.vandewalle@uliege.be
Olivier Collignon, Institut de Recherche en Sciences Psychologiques (IPSY), Université catholique de Louvain, Place Cardinal Mercier 10, B-1348 Louvain-la-Neuve, Belgium. Email: olivier.collignon@uclouvain.be.

Running head: Light modulates occipital alpha in the blind

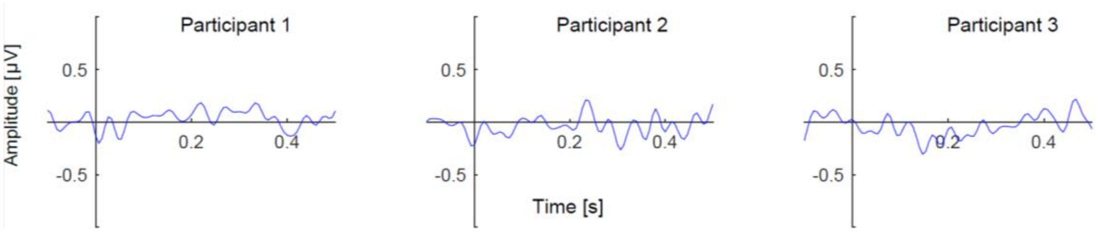

Supplementary Figure S1. Trial averages in the time domain for brief photic stimulation with blue light, relative to baseline showing a marked absence of visual evoked potentials (VEP; 800 trials). Stimulus was delivered at 0.
